# Selected proceedings of Machine Learning in Systems Biology: MLSB 2016

**DOI:** 10.1186/s12859-016-1305-1

**Published:** 2016-12-13

**Authors:** Aalt D. J. van Dijk, Harri Lähdesmäki, Dick de Ridder, Juho Rousu

**Affiliations:** 1 Biometris, Wageningen University and Research, Droevendaalsesteeg 1, 6708 PB Wageningen, The Netherlands; 2 Applied Bioinformatics, Wageningen University and Research, Droevendaalsesteeg 1, 6708 PB Wageningen, The Netherlands; 3 Bioinformatics Group, Wageningen University and Research, Droevendaalsesteeg 1, 6708 PB Wageningen, The Netherlands; 4 Department of Computer Science, Aalto University, 00076 Aalto, Finland

## Introduction

Biology is rapidly turning into an information science, thanks to enormous advances in the ability to observe the molecular properties of cells, organs and individuals. This wealth of data allows us to model molecular systems at an unprecedented level of detail and to start to understand the underlying biological mechanisms. The burgeoning field of systems biology creates a huge need for methods from machine learning, which find statistical dependencies and patterns in these large-scale datasets and use these to establish models of complex molecular systems. MLSB is a successful series of workshops that aims to provide a scientific forum for the exchange between researchers from Systems Biology and Machine Learning, to promote the exchange of ideas, interactions and collaborations between these communities.

MLSB started in 2007 and since 2008 has been co-located with major conferences in computational and systems biology (ECCB 2012, 2014; ISMB/ECCB 2011, 2013; ICSB 2010) or machine learning (ECML 2008–9, NIPS 2015), in order to engage the relevant wider communities. The workshop has constantly attracted around 80 participants or more, 2016 not being an exception: the workshop was fully booked, participant number only limited by the room capacity.

MLSB2016 took place as a two-day pre-conference workshop of the European Conference on Computational Biology, in the Hague, The Netherlands. The focus of the contributions to MLSB ranged from more methodological to more applied, and clearly demonstrated the use of machine learning to address biological questions. Selected submissions were invited based on the papers presented in the workshop. This supplement contains a reviewed selection of six full papers that cover a large panel of topics in Machine Learning devoted to Systems Biology.

Two of the manuscripts [1, 2] deal with the analysis of epigenomic marks. Lukauskas et al. [1] present an approach to cluster and visualize these marks. Their approach adaptively rescales genomic distances in order to enable clustering regions of interest with similar shapes. Park et al. [2] apply association rule mining in order to find differential combinatorial chromatin modification patterns.

Two additional papers describe aspects of (unsupervised) network reconstruction [3, 4]. Affeldt et al. [3] present a consensus method based on spectral decomposition. The basic idea here is to first identify related variables, and then in a second step perform multiple parallel local network reconstructions from which a global network is inferred. The second contribution related to network construction, Heinävaara et al. [4], describes aspects of L1-penalised sparse precision matrix estimation. L1-regularisation is often applied in network reconstruction, and this manuscript demonstrates that it is important to check whether the conditions of consistency are likely to be met by the dataset and the problem at hand. In addition to these two papers focussing on network reconstruction, a third contribution, Veríssimo et al. [5] also deals with networks, using network-based features for regularization in survival analysis. They propose a method that applies network centrality measures to constrain models where the outcome is patient survival and the features are genes. Finally, Gönen [6] presents a Bayesian multiple kernel learning algorithm, which trains a binary classifier with a sparse set of active gene sets using a sparsity-inducing prior. This method is subsequently generalized to a multitask learning setting to model multiple related datasets conjointly.

All in all, the special issue reflects the depth and diversity of data analysis and modelling challenges that the field faces, and the variety of methods that are used to tackle them.
